# Human papillomavirus prevalence and genotype distribution among 30,147 screened women and 3,362 cervical cancer patients in China: a retrospective study

**DOI:** 10.1186/s12985-025-02943-z

**Published:** 2025-10-10

**Authors:** Mei Ye, Dongxia Yan, Jie Xu, Dongqin Liu, Xiaoyan Liu, Wei Jiang, Ling Sha, Ke Long, Yi Feng, Shupeng Zhang, Jun Luo, Hong Xie, Rong Zou, Yuliang Deng, Jianxiang Geng, Jian Huang, Wanqiu Huang

**Affiliations:** 1 Jiangning District Maternal Child Health and Family Planning Service Center, Nanjing, Jiangsu China; 2 Department of Pathology, Maanshan People’s Hospital, Maanshan, Anhui China; 3 Key Laboratory of Systems Biomedicine (Ministry of Education), Shanghai Center for Systems Biomedicine, Shanghai Jiao Tong University, Shanghai, China; 4 Department of Pathology, Yancheng Maternity and Child Health care Hospital, Yancheng, Jiangsu China; 5 Department of Pathology, The Second Affiliated Hospital of Shandong First Medical University, Taian, Shandong China; 6 Reproductive Testing Quality Control, Department of Central Laboratory, Jiangsu Health Vocational College, Nanjing, Jiangsu China; 7 Department of gynecology, Shenzhen People’s Hospital, 2nd Clinical Medical College of Jinan University, Shenzhen, Guangdong China; 8 Department of Pathology, The Affiliated Nanjing Traditional Chinese Medical Hospital of Nanjing Traditional Chinese Medical University, Nanjing, Jiangsu China; 9 The Cross-Strait Precision Medicine Association - HPV Infection Disease Professional Commitee, Nanjing, China

**Keywords:** Cervical cancer screening, Human papillomavirus, HPV genotypes, Age-specific prevalence, HPV-related anxiety, Squamous cell carcinoma, Adenocarcinoma, China

## Abstract

**Background:**

Persistent infection with high-risk human papillomavirus (HR-HPV) is the primary cause of cervical cancer. Understanding genotype distribution and evaluating screening strategies are essential for effective prevention.

**Methods:**

We retrospectively analyzed cervical cancer screening data from 97,686 women aged 35–64 years in Nanjing (2021–2023). Among these, 30,147 underwent combined cytology and HPV testing and 67,539 TCT alone. HPV genotyping was further performed in 3,362 histologically confirmed cervical cancer cases (3,014 squamous cell carcinomas [SCC] and 348 adenocarcinomas [ADC]) from multiple regions in China.

**Results:**

Combined screening achieved a significantly higher detection rate of abnormalities than TCT alone (13.70% vs. 1.79%, *p* < 0.001). Overall HPV positivity was 11.19%, increasing with age and peaking at 16.85% in women aged 60–64. The most frequent genotypes were HPV52, HPV58, and HPV16. In cervical cancer cases, HPV was detected in 92.73% of SCC and 59.77% of ADC. The proportion of HPV-negative cancers increased with age, particularly in ADC.

**Conclusions:**

Combined TCT and HPV testing improves detection of cervical lesions compared with cytology alone. The observed age-specific and histology-specific differences in HPV prevalence and genotype distribution emphasize the need for tailored screening strategies, particularly for older women. These findings provide region-specific evidence to support the refinement of cervical cancer prevention and control strategies in China, particularly in contexts with similar demographic and epidemiological characteristics to the study population.

**Supplementary Information:**

The online version contains supplementary material available at 10.1186/s12985-025-02943-z.

## Background

Cervical cancer (CC) remains one of the leading causes of cancer-related morbidity and mortality among women worldwide, particularly in low- and middle-income countries. According to GLOBOCAN 2022 estimates, there were approximately 662,301 new cases and 348,874 deaths globally, making CC the fourth most common malignancy in women [1]. In China, CC ranks fifth in cancer incidence among women, with 150,700 new cases and 55,700 deaths annually, and the burden is steadily increasing [2]. The Surveillance, Epidemiology, and End Results (SEER) database indicates that the 5-year relative survival rate is 91% for early-stage CC, but declines to 60% for regional disease and 19% for distant metastases [3], underscoring the critical importance of early detection.

In response to the global burden of CC, the World Health Organization (WHO) launched the Global Strategy to Accelerate the Elimination of Cervical Cancer as a Public Health Problem in 2020 [4]. In line with this initiative, China has implemented a national screening program since 2009 targeting women aged 35–64 years, primarily in rural areas, using cytology or human papillomavirus (HPV) testing. The national goal is to reach 50% screening coverage and a 90% early diagnosis rate by 2025 [5, 6]. Further efforts were outlined in the 2023 “Action Plan to Accelerate the Elimination of Cervical Cancer,” emphasizing HPV vaccination, widespread screening, and timely intervention for precancerous lesions.

Persistent infection with high-risk human papillomavirus (HR-HPV)—a non-enveloped DNA virus with over 200 genotypes—is the primary cause of cervical intraepithelial neoplasia and invasive cervical cancer [7–9]. Among these, HPV16, HPV18, and several other HR-HPV types carry the greatest oncogenic potential. While cytology-based screening using the ThinPrep cytologic test (TCT) has been widely implemented, its diagnostic sensitivity can be limited by sampling and interpretation variability [10]. HPV DNA testing offers higher sensitivity and reproducibility [11, 12], but its broader detection spectrum, especially among younger women with transient infections, may lead to overdiagnosis and psychological distress [13].

To address these limitations, co-testing with cytology and HPV has been shown to improve the detection of cervical abnormalities while mitigating the drawbacks of either method alone [14]. By combining molecular and morphological screening, this approach enhances both sensitivity and specificity across different stages of disease progression [15, 16]. However, screening effectiveness can vary significantly across regions due to differences in HPV genotype prevalence, vaccination coverage, and health system capacity. As China’s population continues to age, regionally tailored screening strategies are urgently needed to ensure equitable and effective cervical cancer prevention.

This study evaluates the effectiveness of single TCT versus combined TCT and HPV testing in a large-scale community-based cervical cancer screening program in Nanjing, China. It also characterizes HPV genotype distribution and age-specific infection trends in both screened women and confirmed cervical cancer cases. The study provides evidence to inform screening policies and HPV vaccination strategies, particularly in regions with limited resources and aging populations in China.

## Materials and methods

### Study population and samples collection

This retrospective observational study was conducted from 2021 to 2023 as part of a community-based cervical cancer screening program in Nanjing, Jiangsu Province, China. Eligible participants were women aged 35–64 years who had a history of sexual activity, resided in Nanjing, and provided written informed consent. Exclusion criteria included a history of hysterectomy, chemotherapy, or radiotherapy; current pregnancy, lactation, or menstruation; recent use of hormonal or immunosuppressive agents; or inadequate sample quality. Of the total screened population, 97,686 women met inclusion criteria. Among these, 67,539 underwent cytology (TCT) alone, while 30,147 received combined TCT and HPV testing. Individuals with abnormal or suspicious results were referred for colposcopy and biopsy for diagnostic confirmation.

### Cervical cell collection

Participants were instructed to avoid vaginal douching, sexual activity, or medication use for three full days prior to sampling, and to attend during the non-menstrual period. Trained gynecologists collected ectocervical and endocervical cells using a broom‑type sampler (ThinPrep cervical brush, Hologic Inc., Marlborough, MA, USA). After removing excess mucus, the sampler was rotated five full turns at the external os. The brush head was immediately rinsed into ThinPrep PreservCyt Solution (Hologic Inc., Marlborough, MA, USA), following the protocol; vials were tightly capped, bar‑coded, stored at 4 °C, and transported under cold‑chain conditions to the laboratory within 72 h.

### Cytology testing

Liquid‑based cytology (LBC) slides were prepared on an automated processor (ThinPrep 2000, Hologic Inc., Marlborough, MA, USA) strictly following the manufacturer’s instructions for use. Slides were screened by certified cytotechnologists and independently verified by board‑certified cytopathologists. Reporting adhered to The Bethesda System (TBS, 2014 update), with categories including: negative for intraepithelial lesion or malignancy (NILM); squamous epithelial abnormalities (ASC‑US, ASC‑H, LSIL, HSIL, and SCC); glandular epithelial abnormalities (AGC‑NOS, AGC‑N, AIS, and adenocarcinoma); and other malignancies. Specimen adequacy and ancillary comments were recorded per TBS criteria.

### HPV testing

HPV DNA was extracted from cervical samples using the DP340 nucleic acid extraction kit (Tiangen Biochemical Technology Co., Ltd., Technology, Beijing, China). Quality and concentration of DNA were verified via spectrophotometry (A260/A280 ratio ≥ 1.8) or fluorometry prior to genotyping. HPV genotyping was performed using the Yaneng Bioscience HPV kit (Shenzhen Yaneng Bioscience Co., Ltd., Shenzhen, China), capable of detecting 17 high‑risk HPV (HR‑HPV) types (16, 18, 26, 31, 33, 35, 39, 45, 51, 52, 53, 56, 58, 59, 66, 68, 82) and 10 low‑risk HPV types (6, 11, 40, 42, 43, 44, 55, 61, 81, 83). This assay employs real‑time PCR with fluorescent probes to reliably distinguish HPV genotypes, a method recognized for its high sensitivity and specificity in clinical use. Amplification was conducted on an ABI 7500 Real-Time PCR System (Applied Biosystems, Foster City, CA, USA) following the manufacturer’s protocol.

### Cervical cancer samples

Paraffin-embedded tumor specimens were collected from 3,362 patients who underwent surgery for cervical cancer between 2010 and 2023 across 34 cities in six provinces in China. The cohort included 3,014 cases of squamous cell carcinoma (SCC) and 348 adenocarcinomas (ADC), with median patient ages of 53 (IQR 46–61) and 51 years (IQR 45–59), respectively.

### Histopathologic processing and diagnosis

Resected specimens were immersed in 10% neutral-buffered formalin (Beijing Solarbio Science & Technology Co., Ltd., Beijing, China) following recommended protocols. Tissue processing, including dehydration, clearing, and paraffin embedding, was conducted using an automated tissue processor (Leica TP1020, Leica Biosystems, Wetzlar, Germany) and paraffin embedding station (Leica EG1150, Leica Biosystems, Wetzlar, Germany). Serial sections of 4–5 μm thickness were cut using a rotary microtome (Leica RM2235, Leica Biosystems, Wetzlar, Germany). Sections were stained with hematoxylin and eosin (H&E) using standard reagents (Beijing Solarbio Science & Technology Co., Ltd., Beijing, China) and evaluated under a light microscope (Olympus BX43; Olympus Corporation, Tokyo, Japan). Diagnosis adhered to the 2014 WHO Classification of Tumors of Female Reproductive Organs (4th Edition), which provides a globally standardized framework for tumor classification.

### Statistical analysis

All analyses were conducted using Microsoft Excel (Microsoft Corporation, USA), R version 4.3.1, and GraphPad Prism version 10.2.0. Categorical variables, such as HPV positivity and lesion detection rates, were compared using Pearson’s χ² test or Fisher’s exact test when appropriate. Statistical significance was set at *p* < 0.05. Data visualization was performed using GraphPad Prism to ensure clarity and consistency of result presentation.

## Results

### Cervical disease distribution and screening outcomes

**Fig. 1:**
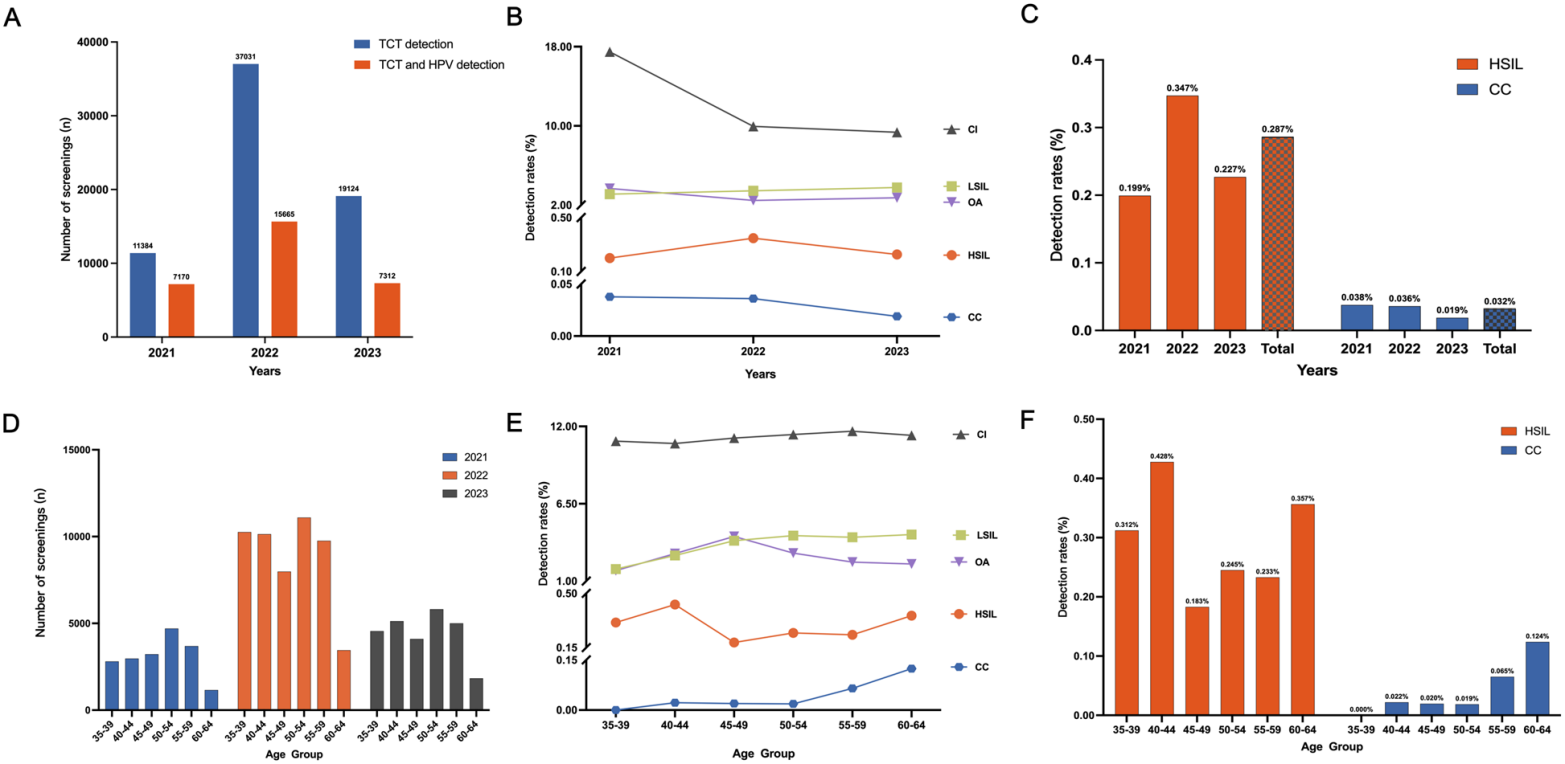
Cervical disease detection rates and demographic distribution in the screened community. (**A**) Annual cervical cancer screening numbers using TCT alone or combined TCT and HPV testing from 2021 to 2023. (**B**) Trends in detection rates of chronic inflammation (CI), low-grade squamous intraepithelial lesion (LSIL), high-grade squamous intraepithelial lesion (HSIL), cervical cancer (CC), and other abnormalities (OA) over three years. (**C**) Annual detection rates of HSIL and CC. (**D**) Age distribution of screened individuals. (**E**) Age-specific detection rates of different diagnostic categories. (**F**) Age-specific detection rates of HSIL and CC

Between 2021 and 2023, 97,686 women aged 35–64 years underwent cervical cancer screening, with 67,539 receiving TCT alone and 30,147 undergoing combined TCT and HPV testing (Fig. 1A). Women with abnormal results underwent further histopathological evaluation, clinical examination, and medical history review.

Among all participants, 82.22% (80,319/97,686) were classified as normal, 11.22% (10,961/97,686) had chronic inflammation, 3.47% (3,387/97,686) had LSIL, 2.77% (2,708/97,686) had other abnormalities, 0.29% (280/97,686) had HSIL, and 0.03% (31/97,686) were confirmed as CC (Table S1).

Over the three-year period, cervical cancer detection exhibited a slight but non-significant decline (*p* > 0.05). HSIL cases peaked in 2022 and were significantly higher than in 2021 and 2023 (*p* < 0.05) (Fig. 1B and C). LSIL incidence showed a rising trend, while the prevalence of chronic inflammation and other abnormalities progressively decreased (*p* < 0.05) (Fig. 1B). These patterns suggest dynamic shifts in disease prevalence that may reflect both screening strategies and underlying population characteristics.

The median participant age was 49 years (IQR: 41–55), with the highest screening uptake among women aged 50–54 (Fig. 1D). CC detection significantly increased with age, reaching 0.080% in women aged 55–64 compared with 0.015% in women aged 35–54 (*P* < 0.05, Table S2). HSIL peaked in women aged 40–44 and 60–64, while LSIL and chronic inflammation rates rose progressively with age (Fig. 1E and F). These findings underscore the importance of age-stratified screening strategies and timely intervention, particularly for older women at elevated risk.

### Comparison between TCT alone and combined TCT + HPV testing

Among 67,539 women undergoing TCT alone, the positivity rate was 1.79% (1,207/67,539), whereas the combined TCT and HPV screening group had a positivity rate of 13.70% (4,131/30,147), with 2.46% (741/30,147) testing positive for both methods and 11.24% (3,390/30,147) for either method alone (Table 1). This indicates that combined screening provides a higher detection yield compared with cytology alone.

**Table 1:** Detection rates of CC and cervical disease across screening methods

Detection methods		CC (*n*, %)	HSIL (*n*, %)	LSIL (*n*, %)	CI (*n*, %)	OA (*n*, %)	Normal (*n*, %)	Total (*n*, %)
TCT	TCT-	1 (0.0015)	1 (0.0015)	1997 (2.96)	7110 (10.53)	1225 (1.81)	55,998 (82.91)	66,332 (98.21)
	TCT+	9 (0.0133)	100 (0.1481)	100 (0.15)	294 (0.44)	43 (0.06)	661 (0.98)	1207 (1.79)
	Total	10 (0.0148)	101 (0.1495)	2097 (3.10)	7404 (10.96)	1268 (1.88)	56,659 (83.89)	67,539 (100.00)
TCT + HPV	TCT- & HPV-	0 (0.0000)	0 (0.0000)	976 (3.24)	2652 (8.80)	856 (2.84)	21,532 (71.42)	26,016 (86.30)
	TCT- & HPV+	7 (0.0232)	34 (0.1128)	99 (0.33)	478 (1.59)	544 (1.80)	1470 (4.88)	2632 (8.73)
	TCT+ & HPV-	1 (0.0033)	12 (0.0398)	59 (0.20)	122 (0.40)	32 (0.11)	532 (1.76)	758 (2.51)
	TCT+ & HPV+	13 (0.0431)	133 (0.4412)	156 (0.52)	305 (1.01)	8 (0.03)	126 (0.42)	741 (2.46)
	Total	21 (0.0697)	179 (0.5938)	1290 (4.28)	3557 (11.80)	1440 (4.78)	23,660 (78.48)	30,147 (100.00)

CC, cervical cancer; HSIL, high-grade squamous intraepithelial lesion; LSIL, low-grade squamous intraepithelial lesion; CI, chronic inflammation; OA, other abnormalities

In TCT alone group, the detection rates for TCT-positive CC and HSIL were 0.0133% (9/67,539) and 0.1481% (100/67,539), respectively. TCT-negative CC and HSIL cases were detected at lower rates of 0.0015% (1/67,539) each. Notably, these two TCT-negative cases were initially identified through prior gynecological examinations, which revealed cervical lesions and polyps, subsequent colposcopy with histopathology confirmed the diagnoses, highlighting the limitations of cytology as a standalone tool.

Combined TCT and HPV screening exhibited higher detection rates for CC (0.0697%, 21/30,147) and HSIL (0.5938%, 179/30,147). Dual-positive cases (both TCT and HPV positive) accounted for 0.0431% (13/30,147) of CC and 0.4412% (133/30,147) of HSIL, while single-positive cases (either TCT or HPV positive) represented 0.0265% (8/30,147) and 0.1526% (46/30,147), respectively.

### HPV genotype distribution in screened women

**Fig. 2:**
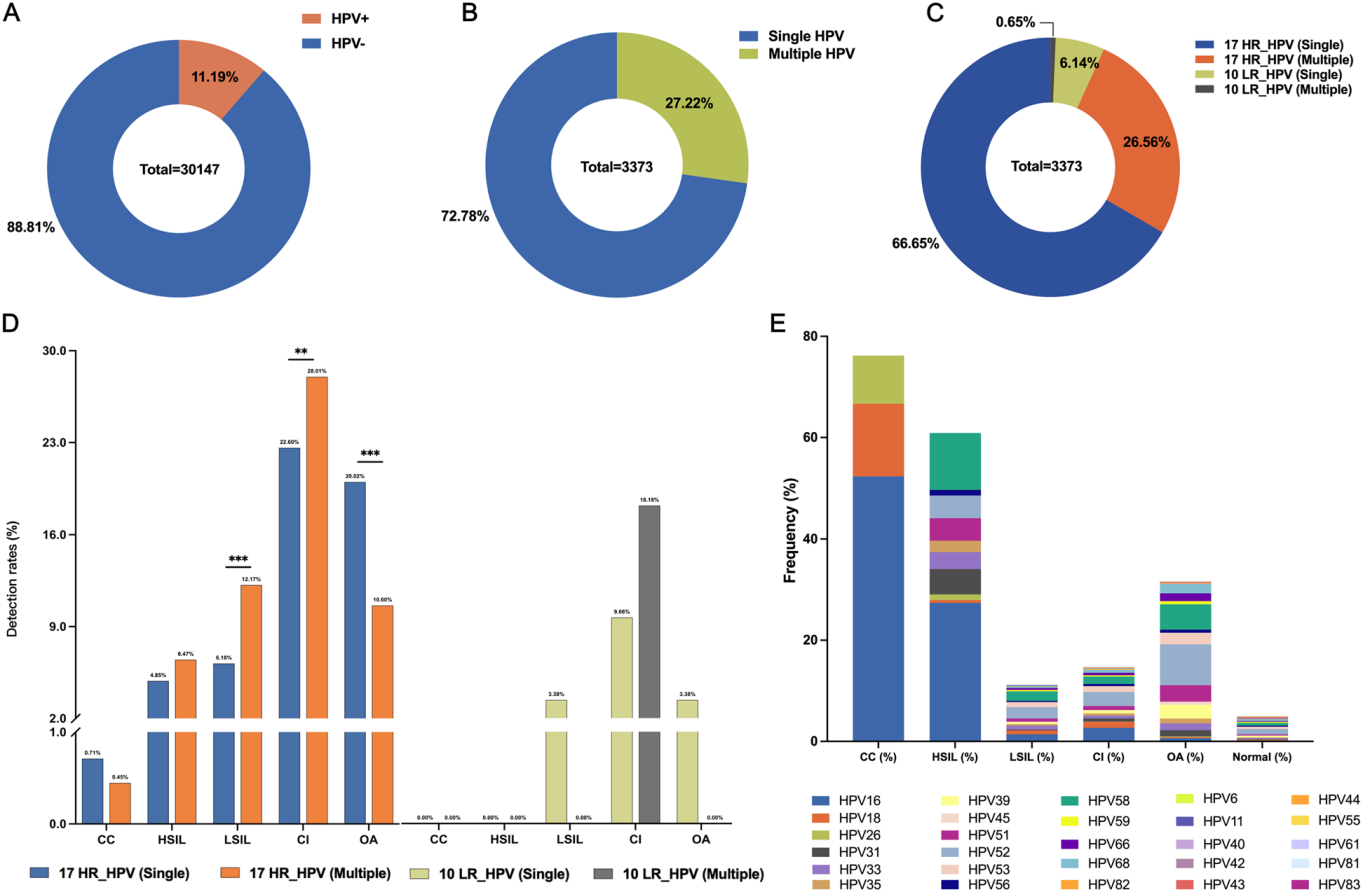
HPV positivity and genotype distribution across cervical disease categories (**A**) Overall HPV positivity rates among 30,147 screened individuals. (**B**) Distribution of single and multiple HPV infections among HPV-positive cases (*n* = 3,373). (**C**) Distribution of single and multiple infections by 17 HR-HPV and 10 LR-HPV genotypes among HPV-positive cases. (**D**) Detection rates of cervical disease categories among across single and multiple HR-HPV and LR-HPV infections. (**E**) Prevalence of specific HPV single genotypes across different cervical disease categories

Among 30,147 women undergoing combined screening, HPV positivity was 11.19% (3,373/30,147) (Fig. 2A). Single-genotype infections predominated (72.78%, 2,455/3,373) (Fig. 2B). Single-genotype HR-HPV infections comprised 66.65% of cases, while LR-HPV infections were rare (6.14% single, 0.65% multiple) (Fig. 2C). Given the dominance of HR-HPV, particularly single-genotype infection, subsequent analyses focused on single-genotype HR-HPV infections.

HPV positivity increased with lesion severity, from 19.77% (255/1,290) in LSIL to 93.30% (167/179) in HSIL and 95.24% (20/21) in CC (Table 2). Within the single HR-HPV infection group, the proportions of CC and HSIL were 0.71% (16/2,248) and 4.85% (109/2,248), respectively, while in multiple-genotype infections, CC and HSIL were observed in 0.45% (4/896) and 6.47% (58/896) (Table S3). No statistically significant differences were observed in the risk of CC or HSIL between single and multiple HR-HPV infections (*P* > 0.05) (Fig. 2D).

**Table 2:** HPV positivity and cervical disease detection rates in the screened population

HPV results	Normal (*n*, %)	OA (*n*, %)	CI (*n*, %)	LSIL (*n*, %)	HSIL (*n*, %)	CC (*n*, %)	Total (*n*, %)
HPV+	1596 (6.75)	552 (38.33)	783 (22.01)	255 (19.77)	167 (93.30)	20 (95.24)	3373 (11.19)
HPV-	22,064 (93.25)	888 (61.67)	2774 (77.99)	1035 (80.23)	12 (6.70)	1 (4.76)	26,774 (88.81)
Total	23,660 (78.48)	1440 (4.78)	3557 (11.80)	1290 (4.28)	179 (0.59)	21 (0.07)	30,147 (100.00)

CC, cervical cancer; HSIL, high-grade squamous intraepithelial lesion; LSIL, low-grade squamous intraepithelial lesion; CI, chronic inflammation; OA, other abnormalities

The most common single-genotype infections in the screened population were HPV52 (1.68%, 505/30,147), HPV58 (0.98%, 294/30,147), and HPV16 (0.75%, 227/30,147). In CC cases, HPV16 was predominant (52.38%, 11/21), followed by HPV18 (14.29%, 3/21) and HPV26 (9.52%, 2/21) (Table S4, Fig. 2E). These findings indicate genotype-specific associations with lesion severity.

### Age-related trends in HPV positivity and genotype distribution

**Fig. 3:**
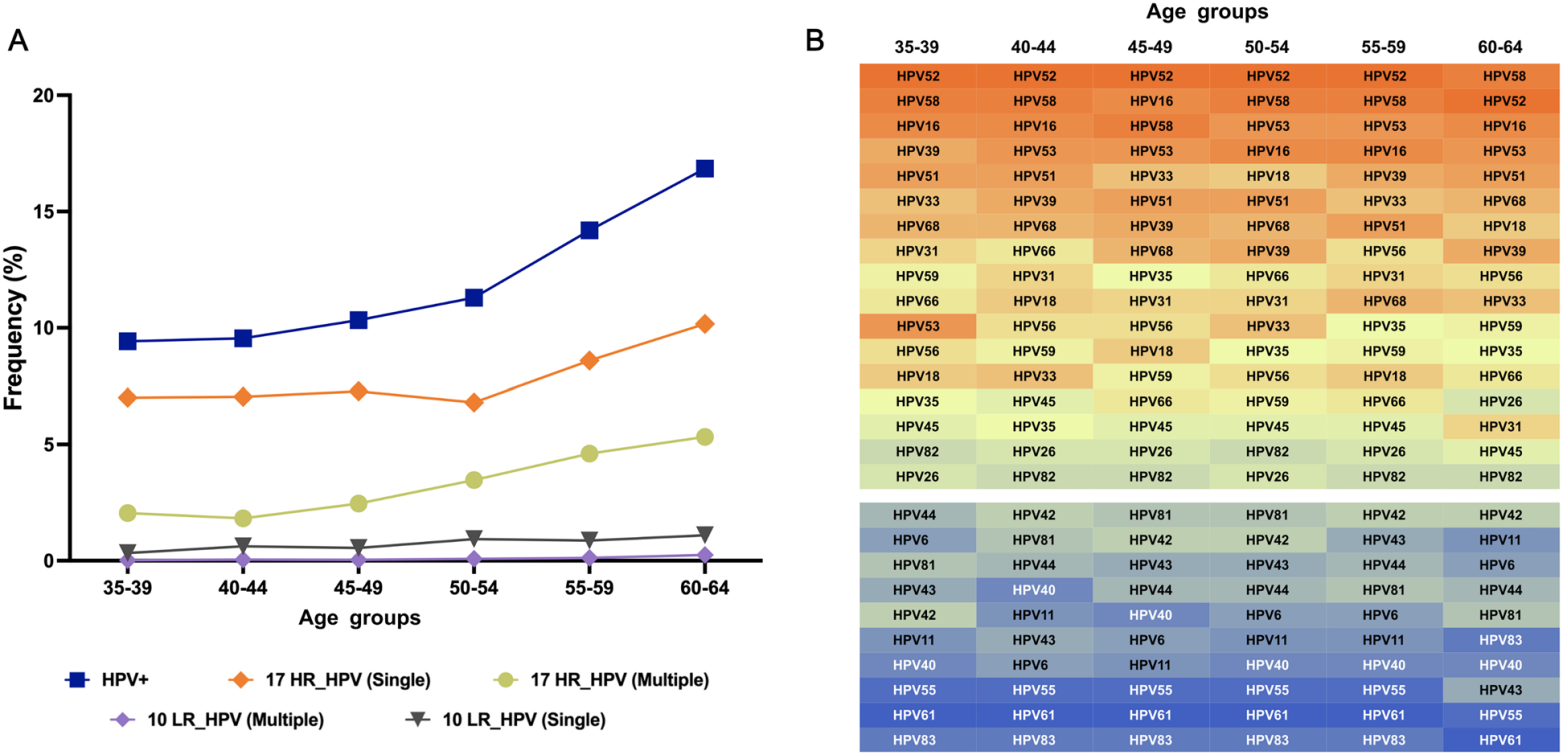
Age-specific trends in HPV positivity and genotype distribution (**A**) HPV positivity rates and distribution of single and multiple infections of 17 HR-HPV and 10 LR-HPV genotypes across age groups. (**B**) Heatmap of single-genotype infection prevalence for 17 HR-HPV and 10 LR-HPV across age groups

In the screening cohort, HPV positivity showed a progressive increase with age, rising from 9.42% in women aged 35–39 years to 16.85% in those aged 60–64 years. This trend was consistent across single and multiple infections with both HR-HPV and LR-HPV genotypes (Fig. 3A, Table S5), indicating a greater burden of infection in older women.

Among single-genotype HPV infections, HPV52, HPV58, and HPV16 were the most prevalent across all age groups. HPV52 dominated among individuals aged 35–59, while HPV58 was most frequent in those aged 60–64. In contrast, HPV26, HPV45, and HPV82 remained rare across all ages (Fig. 3B, Table S6). The seven HR-HPV genotypes (HPV16, 18, 31, 33, 45, 52, and 58) covered by the nine-valent vaccine were detected across all age groups, though HPV45 remained rare. LR-HPV genotypes HPV44, HPV42, and HPV81 were more common than HPV6 and HPV11. These findings emphasize the importance of HPV vaccination and age-stratified screening strategies for effective cervical cancer prevention.

### HPV positivity in cervical cancer cases

**Fig. 4:**
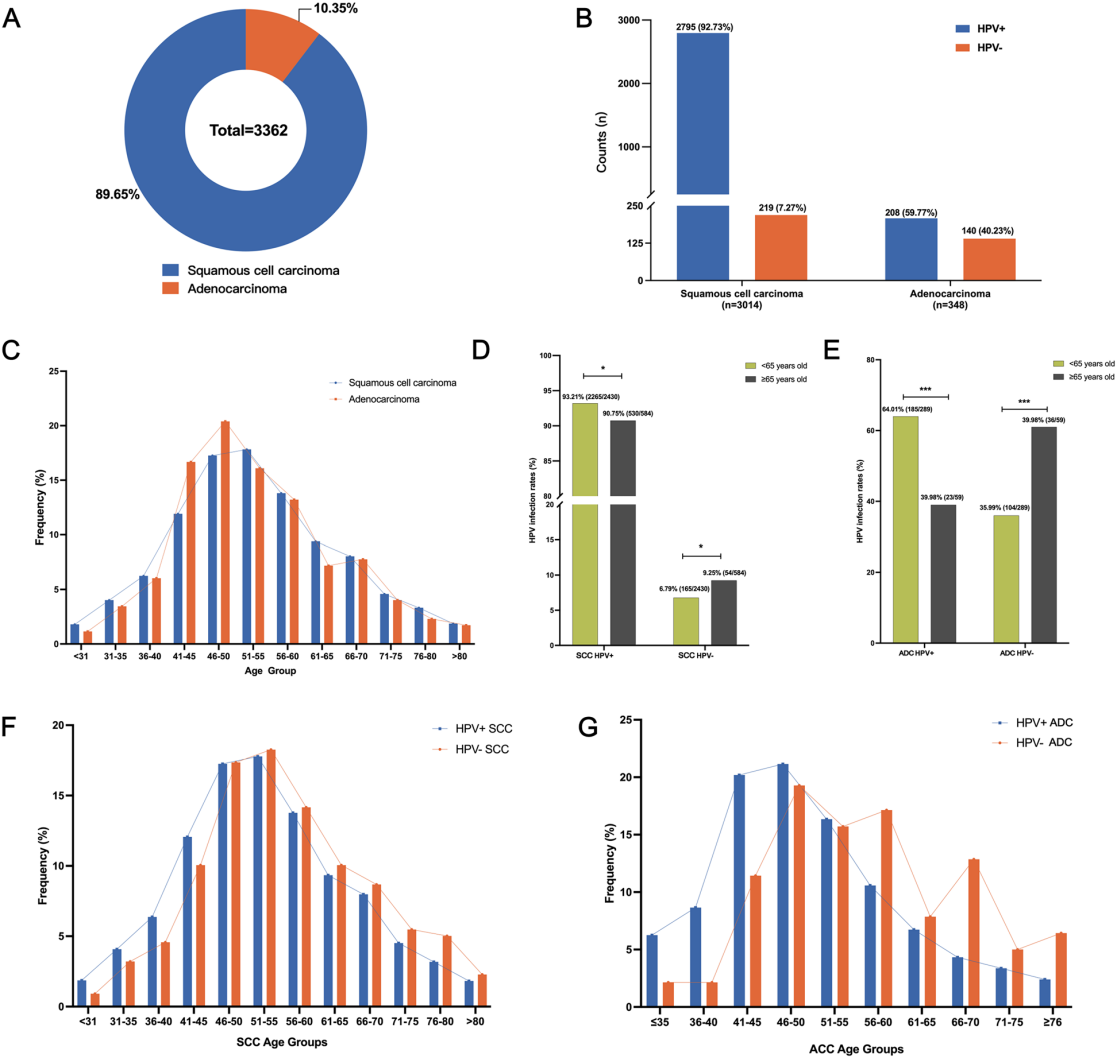
HPV positivity and age-related patterns in cervical cancer samples. (**A**) Proportions of SCC and ADC among 3,362 cervical cancer cases. (**B**) HPV positivity rates in SCC and ADC. (C) Age distribution of SCC and ADC patients. (**D**) HPV positivity in SCC patients aged < 65 and ≥ 65 years. (**E**) HPV positivity in ADC patients aged < 65 and ≥ 65 years. (**F**) Age distribution within HPV-positive and HPV-negative SCC cases. (**G**) Age distribution within HPV-positive and HPV-negative ADC cases

Given only 31 CC cases were detected in the screened community cohort, detailed statistical analysis was limited. To comprehensively assess HPV infection patterns in CC, 3,362 CC tissue samples were collected from multiple hospitals across China, comprising 3,014 SCC and 348 ADC cases (Fig. 4A). Overall HPV positivity was significantly higher in SCC (92.73%) compared with ADC (59.77%) (Fig. 4B, Table S7).

Age distribution patterns differed slightly between subtypes. SCC incidence peaked at 51–55 years, whereas ADC peaked at 46–50 years before declining in older groups (Fig. 4C). HPV positivity decreased with age in both subtypes. In SCC, HPV detection was 93.21% in patients under 65 years compared with 90.75% in those over 65 (*P* < 0.05) (Fig. 4D). In ADC, positivity was 64.01% in women under 65 years but dropped to 39.98% in those over 65 (*P* < 0.05) (Fig. 4E).

To further examine age distribution by HPV status, the proportion of each age group was calculated within the HPV-positive and HPV-negative populations of SCC and ADC (Fig. 4F–G, Tables S8–S9). In SCC before 45 years, the proportion of each age group within the HPV-positive SCC population was higher than within the HPV-negative SCC population, but this trend reversed after 45 years, with HPV-negative SCC cases becoming more prevalent (Fig. 4F, Table S8). In ADC, HPV-positive cases were more prevalent before 55 years, but HPV-negative cases dominated after 55 years (Fig. 4G, Table S9). These patterns indicate an age-related decline in HPV positivity and a rising proportion of HPV-negative CC in elderly women.

### HPV genotype distribution in HPV-positive cervical cancer

**Fig. 5:**
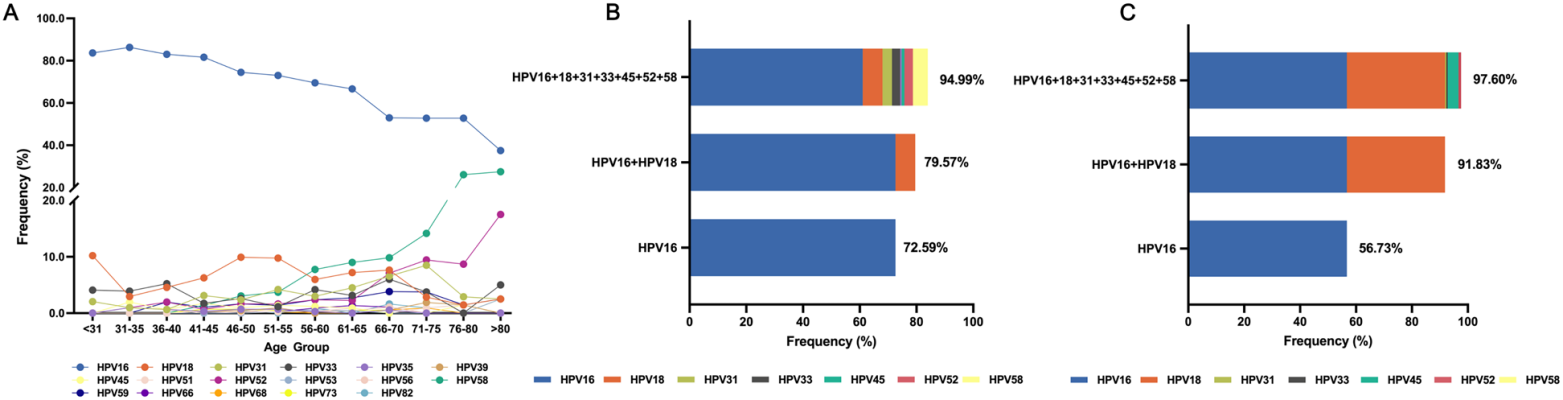
HPV genotype distribution in HPV-positive cervical cancer patients. (**A**) Age-specific distribution of HPV genotypes in HPV-positive SCC cases. (**B**) Cumulative detection rates of HPV16 alone, HPV16 + HPV18, and seven HR-HPV genotypes in HPV-positive SCC. (**C**) Cumulative detection rates of HPV16, HPV16 + HPV18, and seven HR-HPV genotypes in HPV-positive ADC

In SCC, all single-genotype infections were HR-HPV types, with HPV16 (56.60%), HPV18 (5.71%), and HPV58 (4.68%) being the most prevalent. HPV16 positivity declined with age, whereas HPV58, HPV52, HPV31, and HPV82 showed modest increases (Fig. 5A; Table S10). In ADC, HPV16 (51.18%), HPV18 (39.41%), and HPV45 (4.71%) were dominant, although no consistent age-related pattern was observed (Table S11).

Among HPV-positive CC cases, HPV16 was detected in 72.59% of SCC and 56.73% of ADC cases, with HPV16/18 together accounting for 79.57% of SCC and 91.83% of ADC. The seven HR-HPV genotypes included in the nine-valent vaccine were present in 94.99% of SCC and 97.60% of ADC cases (Fig. 5B and C). These findings indicate that most HPV-positive CC in this cohort were attributable to vaccine-covered genotypes, underscoring the relevance of current vaccination strategies, detecting these HR-HPV types could cover a large proportion of HPV-positive CC cases.

## Discussion

This study demonstrates that combined HPV and cytology screening yields substantially higher detection rates of cervical lesions than cytology alone (13.70% vs. 1.79%, Table 1). The improved performance reflects the complementary strengths of the two methods: HPV testing identifies viral infection before morphological abnormalities emerge, whereas cytology detects cellular changes once they occur [17]. By integrating these approaches, combined screening enhances sensitivity and reduces the risk of false negatives [10, 18]. These results suggest that broader adoption of combined screening could support earlier detection and potentially reduce CC burden at the population level [15, 16].

In addition, our findings highlight regional variation in HPV prevalence. Nationally, the pooled HPV prevalence in China is estimated at 15.54% (HR-HPV: 11.93%, LR-HPV: 2.78%) [19]. The overall HPV positivity rate in Nanjing was 11.19%, which was similar to reports from Suzhou (10.17%) but lower than in Beijing (12.18%), Xinjiang (14.02%), Zhejiang (15.5%), Yunnan (16.02%), and Henan (19.7%), while higher than Tibet (8.16%) [20–29]. Globally, prevalence varies even more widely, from 26.1% in sub-Saharan Africa to 2.6% in North America and 4.4% in Europe [30]. These geographic differences may reflect variations in sexual behavior, access to healthcare, hygiene standards, and host genetic susceptibility [31–36]. For example, early sexual debut or multiple partners elevate HPV risk, while comprehensive sexual education and screening programs mitigate infection rates. Limited healthcare access and poor hygiene can exacerbate viral transmission, whereas genetic polymorphisms may shape host immune responses [31–36]. Taken together, these findings underscore the importance of tailoring HPV prevention and screening strategies to local epidemiological and social contexts.

This study revealed important distinctions between HPV-positive and HPV-negative SCC. Although HPV-positive cases greatly outnumber HPV-negative ones, their age distributions were broadly similar, with incidence peaking between 45 and 55 years. This pattern suggests that factors beyond HPV infection may contribute to cervical carcinogenesis, including menopausal changes and chronic inflammation. Previous studies have reported that declining estrogen levels during menopause can impair cervical epithelial integrity, increasing vulnerability to inflammation and other external influences such as sexual activity and microbiota imbalance [37, 38].

HPV-negative cervical cancer exhibits distinct clinical and molecular features that differ from HPV-positive disease. Clinically, HPV-negative tumors are associated with poorer prognosis, earlier lymph node metastasis, and reduced responsiveness to radiotherapy and immunotherapy [39, 40]. At the molecular level, HPV-negative cases often display lower proliferative activity, increased p53 expression, downregulated p16, and higher frequencies of KRAS, ARID1A, and PTEN mutations. By contrast, HPV-positive tumors typically show enhanced proliferation, reduced p53 expression, integration of HPV E6/E7, and frequent APOBEC mutational signatures [40]. Another notable observation is the age-associated variation in HPV positivity. In our cohort, HPV prevalence was highest in women aged 60–64 years, with 90.75% of SCC cases in women ≥ 65 years being HPV-positive. This pattern is consistent with previous reports of a U-shaped age distribution, with elevated rates in both younger and postmenopausal women [19, 41]. The resurgence of HPV in older age groups may be linked to immunosenescence and diminished viral clearance. Although we did not separately analyze cytology outcomes in women > 65 years, the close association between HPV infection and cervical lesions indicates that older women remain at risk for undetected precancerous changes. Current guidelines, including those from China and the U.S. Preventive Services Task Force, recommend continued screening beyond age 65 under certain conditions such as insufficient prior testing or clinical indications [42, 43]. Given that older women often present with more advanced disease due to missed screening or asymptomatic progression [44–46], expanding coverage in this population could reduce disease burden and mortality through earlier intervention. 

In this study, the HPV positivity rate in SCC (92.73%) was consistent with previously reported ranges of 90–95% [47–49], reinforcing the role of HR-HPV, particularly HPV16, as a predominant etiological factor. By contrast, ADC showed a lower positivity rate (59.77%), below the 70–80% typically reported [50–52], suggesting that additional non-HPV-related mechanisms may contribute to its pathogenesis [53]. Among SCC cases with single-genotype infections, HPV16 was the most common (71.08%), followed by HPV18 (7.17%) and HPV58 (5.88%), consistent with earlier reports [47]. Notably, the prevalence of HPV16 declined with increasing age, whereas HPV58, HPV52, HPV31, and HPV82 became more frequent. In ADC, single-genotype infections were dominated by HPV16 (51.18%) and HPV18 (39.41%), with HPV45 (4.71%) also detected, consistenting with previous regional studies [50–52]. These findings underscore the distinct epidemiological and etiological profiles of SCC and ADC, highlighting the need for histology-specific screening and prevention strategies.

From a prevention perspective, the genotype distribution observed here further supports the relevance of current vaccines. In the screened population, the overall HPV positivity rate was 11.19%, of which HR-HPV accounted for 10.43%, and the seven HR-HPV genotypes included in the nine-valent vaccine represented 6.72% of all infections, while HPV16/18 accounted for 1.77%. Among SCC cases, 94.99% of HPV-positive samples harbored these seven HR-HPV types, with 79.57% positive for HPV16/18 and 72.59% for HPV16 alone. In ADC, the corresponding proportions were 97.60%, 91.83%, and 56.73%. These results are consistent with prior studies showing that HPV16/18 account for 70.8% of cervical cancers, and that the seven HR-HPV types targeted by the nine-valent vaccine collectively explain nearly 90% of cases [54]. These findings suggest that vaccination strategies focusing on these high-risk genotypes can provide broad protection against HPV-positive cervical cancers.

The oncogenic potential of HPV varies substantially by genotype. According to the International Agency for Research on Cancer (IARC), Group 1 carcinogenic types include HPV16, 18, 31, 33, 35, 39, 45, 51, 52, 56, 58, and 59; Group 2 A (probably carcinogenic) includes HPV26, 53, 66, 73, and 82; and Group 2B (possibly carcinogenic) includes HPV34, 67, 68, 69, 70, 85, and 97 [55]. While broader genotyping improves the sensitivity of early lesion detection, it may also generate unintended consequences. Many women misinterpret an HPV-positive result as an imminent cancer risk, which can cause considerable anxiety and reduce quality of life [56–58]. As most infections are transient and only persistent HR-HPV is clinically relevant, clearer health communication and structured interpretation pathways are essential. Public education should emphasize that HPV positivity indicates risk but is not synonymous with malignancy. Integrating targeted screening, HPV vaccination, and optimized follow-up protocols can improve efficiency while minimizing unnecessary psychological harm.

This study has limitations, including limited regional representativeness, a small sample size for women aged ≥ 65 years, and non-randomized allocation to screening methods (cytology alone vs. combined testing), which may introduce selection bias and limit generalizability to different healthcare settings. Future studies will: (1) expand sample coverage through multi-center, cross-regional recruitment, incorporating diverse age groups (particularly women ≥ 65 years) and socioeconomic backgrounds, larger cohorts of HPV-negative and adenocarcinoma cases, with stratification by vaccination status and behavioral risk factors, will enable more comprehensive subgroup analyses and better reflect real-world diversity; (2) integrate health economic evaluations of screening methods and apply molecular techniques to investigate the pathogenesis of HPV-negative cervical cancer. Genotype-specific risk stratification will also be refined by comparing carcinogenic potential and persistence, to inform vaccine development and screening strategies; (3) adopt randomized controlled trial designs to reduce selection bias and more robustly compare screening effectiveness, with long-term follow-up to assess the progression of HPV infection and optimize screening intervals; (4) analyze regional differences in screening coverage and vaccination rates to support localized prevention strategies, and evaluate the protective efficacy of existing vaccines, particularly against prevalent genotypes such as HPV52/58, to guide future vaccine promotion and policy implementation.

## Conclusions

This large-scale study (2021–2023) in Nanjing, China, confirms that combined cytology and HPV testing is more effective than cytology alone for detecting cervical lesions and invasive cancers. The higher HPV positivity in women over 60 emphasizes the need for sustained, age-tailored surveillance in older populations. Distinct genotype distributions between SCC and ADC—with HPV16/18 remaining dominant and HPV52/58 contributing substantially in this region—support histology-specific prevention strategies. These findings highlight the value of region- and age-adapted screening protocols, inclusion of key carcinogenic HPV genotypes in prevention efforts, and improved communication to reduce patient anxiety. Collectively, they provide evidence to inform the refinement of cervical cancer screening and prevention strategies in China and similar epidemiological contexts.

## Supplementary Information

Supplementary Material 1

Supplementary Material 2

Supplementary Material 3

Supplementary Material 4

Supplementary Material 5

Supplementary Material 6

Supplementary Material 7

Supplementary Material 8

Supplementary Material 9

Supplementary Material 10

Supplementary Material 11

## Data Availability

All data supporting the findings of this study are included in the main article and supplementary materials.
